# SY-1530, a highly selective BTK inhibitor, effectively treats B-cell malignancies by blocking B-cell activation

**DOI:** 10.20892/j.issn.2095-3941.2020.0291

**Published:** 2021-07-15

**Authors:** Liao Wang, Yinghui Sun, Xijie Liu, Hongjuan Li, Chang Lu, Ronghui Yang, Chuanzhen Yang, Binghui Li

**Affiliations:** 1 Department of Biochemistry and Molecular Biology, School of Basic Medical Sciences, Capital Medical University, Beijing 100069, China; 2 Advanced Innovation Center for Human Brain Protection, Capital Medical University, Beijing 100069, China; 3 Shouyao Holdings Co., Ltd, Beijing 100195, China

**Keywords:** SY-1530, BTK, irreversible inhibitor, BCR signaling, B-cell malignancies

## Abstract

**Objective:**

B-cell antigen receptor (BCR) signaling is required to maintain the physiological functions of normal B cells and plays an important pathogenic role in B-cell malignancies. Bruton tyrosine kinase (BTK), a critical mediator of BCR signaling, is an attractive target for the treatment of B-cell malignancies. This study aimed to identify a highly potent and selective BTK inhibitor.

**Methods:**

Homogeneous time-resolved fluorescence assays were used to screen BTK inhibitors. Typhoon fluorescence imaging and Western blot analysis were used to confirm the effects of SY-1530 on the BCR signaling pathway. Additionally, the anti-tumor activities of SY-1530 were evaluated in TMD8 xenografts and spontaneous canine B-cell lymphoma.

**Results:**

We found a novel irreversible and non-competitive inhibitor of BTK, SY-1530, which provided dose-dependent and time-dependent inhibition. SY-1530 selectively bound to BTK rather than inducible T-cell kinase; consequently, it did not significantly affect T-cell receptor signaling and caused limited off-target effects. SY-1530 blocked the BCR signaling pathway through down-regulation of BTK activity, thus leading to impaired phosphorylation of BTK and its downstream kinases. Moreover, SY-1530 induced apoptosis in a caspase-dependent manner and efficaciously inhibited tumor growth in mouse xenograft models of B-cell malignancy (*P* < 0.001). SY-1530 also induced positive clinical responses in spontaneous canine B-cell lymphoma.

**Conclusions:**

SY-1530 is an irreversible and selective BTK inhibitor that shows inhibitory effects on B-cell malignancies by blocking the BCR signaling pathway. Therefore, it may be a promising therapeutic approach for the treatment of B-cell malignancies.

## Introduction

B-cell malignancies, including non-Hodgkin’s lymphomas, some leukemias, and myelomas^1,2^, comprise approximately 85% to 90% of all non-Hodgkin’s lymphomas, and are represented by numerous distinct diseases, such as chronic lymphocytic leukemia (CLL), follicular lymphoma, mantle cell lymphoma (MCL), and diffuse large B-cell lymphoma^3–5^. The 5-year relative survival for patients with B-cell malignancies is 62.8%, according to the data from the Surveillance, Epidemiology, and End Results (SEER) Program. Therefore, human B-cell malignancies continue to pose a medical challenge. The pathogenesis of B-cell malignancies is largely based on aberrant activation of the B-cell antigen receptor (BCR) signaling pathway^6,7^. Consequently, many BCR-related kinases have emerged as therapeutic targets for the treatment of B-cell malignancies.

Bruton tyrosine kinase (BTK) is a non-receptor tyrosine kinase that is mutated in the primary immunodeficiency X-linked agammaglobulinemia^8^. It is a member of the Tec family, which plays an essential role in the BCR signaling pathway^9^. The Tec family consists of 5 members: BTK, Tec, BMX, inducible T-cell kinase (ITK), and RLK^10,11^. ITK, RLK, and Tec are expressed in T cells, whereas BTK is expressed in B lymphocytes and myeloid cells^12,13^. BTK is autophosphorylated at the Tyr-223 residue and is activated in response to the engagement of the BCR by antigen^14–16^. It forms a complex with BLNK and subsequently activates phospholipase C-γ2 (PLCγ2), thus leading to calcium mobilization and activation of transcriptional signaling^17–19^. BTK is required for normal B cell development and differentiation, and is associated with the survival and progression of B-cell malignancies^9,20^. In addition, BTK is up-regulated in B-cell malignancies, and BTK inhibitors have been developed to treat these cancers^21–24^.

Ibrutinib, the first-generation BTK inhibitor, selectively binds BTK at the cysteine 481 residue, thus abrogating BTK activity. It inhibits autophosphorylation of BTK at tyrosine 223 and blocks BCR signaling^25^. Ibrutinib abolishes proliferation and induces apoptosis in CLL cells^26^, and it has been approved by the FDA as the first-line treatment for patients with CLL, MCL, and Waldenström’s macroglobulinemia^27–32^. Up to 70% of patients with relapsed/refractory CLL have an objective response to ibrutinib^33^. However, adverse reactions, including pneumonia, atrial fibrillation, bleeding, and rash, have been observed to be associated with ibrutinib, because of its off-target effects on other proteins of the Tec family and the epidermal growth factor receptor^34^. Moreover, drug resistance has been found to emerge in patients with B-cell malignancies who receive ibrutinib^35–37^. Therefore, next-generation BTK inhibitors would provide new targeted therapies for B-cell malignancies.

The second-generation BTK inhibitors, acalabrutinib and zanubrutinib, have been approved by the FDA for hematologic malignancies. Acalabrutinib is a more potent and selective BTK inhibitor with fewer off-target effects than ibrutinib. It is currently under evaluation for the treatment of various hematological malignancies and solid tumors^38,39^. Zanubrutinib has higher BTK specificity than ibrutinib, and exposure to zanubrutinib is higher than acalabrutinib or ibrutinib exposure^40,41^. It is currently being evaluated in phase 2 and phase 3 trials in several B-cell malignancies. However, acalubrutinib and zanubrutinib are also associated with adverse events, including skin rash and bruising^42^. Moreover, these drugs cannot treat ibrutinib-resistant patients with a BTK^C481S^ mutation. Therefore, third-generation BTK inhibitors that effectively target BTK and the BTK^C481S^ mutant are being actively evaluated in clinical trials^43^.

Here, we demonstrate that SY-1530, a novel irreversible and highly potent BTK inhibitor, is more selective and has fewer off-target effects than ibrutinib. SY-1530 blocks BCR signaling, inhibits cell growth in malignant B-cell lymphoma cell lines, and induces apoptosis in a caspase-dependent manner. It also suppresses tumor growth in TMD8 mouse xenografts, and shows significant inhibitory activity in spontaneous canine B-cell lymphoma. Overall, our findings suggest that SY-1530 is a novel selective BTK inhibitor that may be used for the treatment of B-cell malignancies.

## Materials and methods

### Antibodies, reagents, and plasmids

The antibodies to β-actin (5125), poly(ADP-ribose) polymerase (PARP) (9532S), cleaved caspase-3 (9661S), BTK (8547), PLCγ2(55512), ERK1/2 (9107), phospho-PLCγ2 (Tyr1217) (3871), phospho-BTK (Tyr223) (87457), and ERK1/2 pT202/pY204 (4377) were purchased from Cell Signaling Technology. An Annexin V-PE Apoptosis Detection Kit (555763), and PE mouse anti-human CD69 (555531), PE mouse anti-human CD3 (555340), and PE mouse anti-human CD19 (561741) were purchased from BD Bioscience. Dynabeads^®^ Human T-Activator CD3/CD28 (11161D) was purchased from Life Technology. Goat anti-human IgM F(ab)2 (2022) was purchased from SouthernBiotech. An HTRF^®^ KinEASE™-TK Kit (62TK0PEC) was purchased from Cisbio. Fugene HD Transfection Reagent (04709705001) was purchased from Roche. A Pierce™ BCA Protein Assay Kit (23225) was purchased from Thermo Fisher Scientific. Cell-Titer Blue^®^ (G8081) was purchased from Promega. Human wild-type BTK and BTK C481A mutants were amplified by PCR and cloned into the pcDNA3.1 vector. All expression plasmids were verified by DNA sequencing.

### Cell culture and transfection

HEK293T and Jurkat cells were obtained from the ATCC in 2014. DOHH2 and Pfeiffer cells were purchased from Beijing Jinzijing Biomedical Technology in 2014. The cell lines were authenticated through short tandem repeat analysis in 2017 and were examined by PCR to exclude mycoplasma contamination. The cell lines were cultured in DMEM or RPMI-1640 (Gibco) supplemented with 1% penicillin-streptomycin (Gibco) and 10% FBS (HyClone). Cells were transfected with Fugene HD Transfection Reagent according to the manufacturer’s instructions (Roche).

### Homogeneous time-resolved fluorescence assays

Homogeneous time-resolved fluorescence (HTRF) assays were performed with a HTRF^®^ KinEASE™-TK kit (Trevigen). Briefly, the compounds dissolved in DMSO, BTK kinase, ATP (at *K_m_*), and TK peptide were added to 384-well plates at a volume of 10 µL per well and incubated at 23 °C for 120 min. Eu^3+^ cryptate-labeled anti-phosphotyrosine antibody and Streptavidin-XL-665 were added to the mixture after the reaction was stopped. After an additional 1 h incubation, the fluorescence signals (excitation at 320 nm, and emission at 615 and 665 nm) were read with an EnVision multilabel plate reader (PerkinElmer). Half-maximal inhibitory concentration (IC_50_) values of the compounds were generated in GraFit software (Version 6.0) and are presented at the mean of 3 independent experiments performed in duplicate.

### Typhoon fluorescence imaging

Cells were treated as indicated before the addition of a fluorescent probe (FL-SY-1530). After incubation, cells were collected and lysed for 5 min in cell lysis buffer. For analysis of the *in vivo* occupancy of BTK inhibitors, normal BALB/c mice (6–8 weeks old) were orally administered BTK inhibitors, then the spleens were processed to splenocytes 3 h after administration and the splenocytes were seeded on 12-well plates and incubated with the fluorescent probe for 1 h. Then the samples were subjected to SDS-PAGE, and the gels were scanned with a Typhoon imaging system (excitation at 532 nm and emission at 555 nm). After scanning, the gels were detected with Western blot.

### Western blot

After the indicated treatments, cells were washed twice with cold PBS and lysed in buffer (1% NP-40, 20 mM Tris-HCl, pH 8.0, 137 mM NaCl, 10% glycerol, 1 mM sodium orthovanadate, 1 mM PMSF, phosphatase inhibitors, and protease inhibitors) on ice for 5 min. Cell lysates were centrifuged at 12,000 × *g* for 10 min. The supernatant was collected, and quantification of total protein was performed with a BCA protein assay kit. Equal amounts of protein (50 µg) were loaded onto SDS-PAGE gels for Western blot with different antibodies.

### Cell viability assays

Cells were seeded on 96-well plates (9,000 cells/well), cultured at 37 °C in 5% CO_2_ overnight, and incubated with different concentrations of compounds for 3 or 7 days. Then the cells were stained with CellTiter-Blue^®^ reagent (Promega) for 4 h at 37 °C. The fluorescence signals were detected with a FlexStation 3, and the IC_50_ values of each compound were determined with GraFit software (Version 6.0).

### Apoptosis detection

Apoptosis assays were performed with an Annexin V-PE Apoptosis Detection Kit I (BD Biosciences). Briefly, 5 × 10^5^ Pfeiffer cells were seeded on 6-well plates and treated with BTK inhibitors at the indicated concentrations. After 24 h or 48 h, cells were collected and stained with PE Annexin V and 7-AAD according to the manufacturer’s instructions. FACS analysis was performed with a Guava flow cytometer.

### B and T cells

CD19^+^ B (>80% purity) and CD3^+^ T (>90% purity) cells were purified with human B cell enrichment cocktail and human T cell enrichment cocktail (Stem Cell Technology), respectively. Cells were seeded on 12-well plates and incubated with different concentrations of BTK inhibitors for 1 h. B cells were stimulated with 50 µg/mL anti-human IgM F(ab′)_2_, and T cells were stimulated with 50 µg/mL anti-CD3/CD28 beads overnight. In the washout groups, the cell medium containing the indicated inhibitors was replaced with fresh medium before stimulation. Then cells were then incubated with anti-human CD69 for 1 h and analyzed with a Guava flow cytometer.

### Tumor xenograft studies

TMD8 cells (1.0 × 10^7^ cells in 100 µL of serum-free medium containing 40% Matrigel) were injected subcutaneously into SCID mice (6–8 weeks of age), and the tumor volume was monitored twice per week. When the tumor volumes reached 150–200 mm^3^, the mice were randomized to groups (8 mice per group) treated with SY-1530 or ibrutinib for 29 days. The compounds were formulated in 0.5% methylcellulose containing 0.1% sodium lauryl sulfate. Tumor size and body weight were measured twice per week. All experiments were approved by the institutional animal care and use committee of Shouyao Holdings and were performed in accordance with the guidelines in the U.S. National Institutes of Health Guide for the Care and Use of Laboratory Animals (Approval No. CTA-2015063).

### Spontaneous canine lymphoma

Dogs with spontaneous lymphoma were purchased from the China Agricultural University Veterinary Teaching Hospital, and the study was approved by the institutional animal care and use committee of Shouyao Holdings (approval No. CTA-2014003). The inclusion criterion was a confirmed histologic diagnosis of B-cell lymphoma (on the basis of immunohistochemistry for CD21 and CD79a). SY-1530 capsules (20–40 mg) were administered daily until disease progression. The dogs were re-examined every week for 4 weeks and subsequently biweekly. The sum of the longest diameters of all target lesions was defined as the tumor burden. According to the criteria for evaluation of the response of peripheral nodal lymphoma in dogs developed by the Veterinary Cooperative Oncology Group, the response was classified as complete response, partial response, progressive disease, or stable disease.

### Statistical analysis

All results for continuous variables are presented as mean ± SEM (*P < 0.05, **P < 0.01, and ***P < 0.001). Statistical analysis was performed with Student’s *t*-test (two-tailed) and two-way analysis of variance.

## Results

### SY-1530 is a highly selective and non-competitive BTK inhibitor

**Figure 1:**
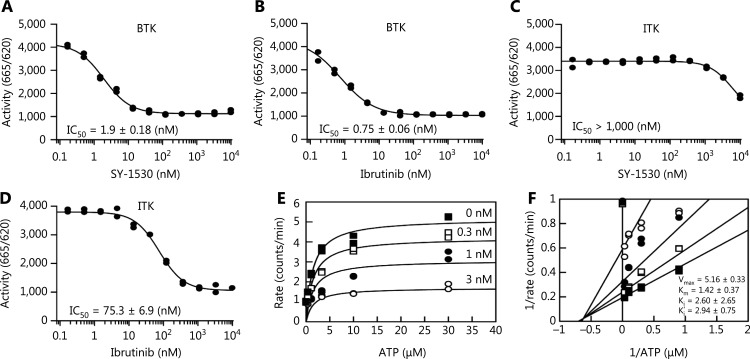
SY-1530 is a highly selective and non-competitive BTK inhibitor. (A–D) The inhibitory effect of SY-1530 or ibrutinib on the activity of BTK or ITK was measured with HTRF assays. Different concentrations of SY-1530 or ibrutinib (gradient dilution of compounds with DMSO) were used, and IC_50_ was calculated with GraFit6.0. (E and F) HTRF assays were performed with different concentrations of SY-1530 (3, 1, 0.3, and 0 nM) and ATP (0–30 μM) to detect the activity of BTK. The enzyme kinetic parameters (K_m_, V_max_, and K_i_) were calculated (E), and the Lineweaver-Burk plot was obtained according to the Michaelis-Menten equation (F).

To identify new BTK inhibitors, we detected the effects of the synthesized compounds on BTK activity by performing HTRF assays. Among the compounds examined, SY-1530 at very low concentrations showed significant inhibitory effects toward BTK activity, thus suggesting that SY-1530 is a potent BTK inhibitor. We next compared SY-1530 with the previously reported BTK inhibitor ibrutinib by measuring the IC_50_ toward BTK activity. SY-1530 had a high BTK inhibitory activity similar to that of ibrutinib (**Figure 1A and 1B**). We subsequently assessed the selectivity of SY-1530 by measuring its inhibition of ITK, which is expressed mainly in T cells. As shown in **Figure 1C and 1D**, the IC_50_ of SY-1530 toward ITK exceeded 1000 nM, a value much greater than that of ibrutinib (75.3 nM), thus suggesting the superiority of SY-1530 over ibrutinib, as it has higher selectivity for BTK than for ITK. Adenosine triphosphate (ATP) is the prosthetic group of protein kinases. According to their interaction with ATP, inhibitors can be classified into 3 types: competitive, noncompetitive, and uncompetitive^44^. The inhibitory effect of SY-1530 on BTK enzymatic activity was not reversed with increasing concentrations of ATP (**Figure 1E**). Moreover, the kinetic parameters showed that the affinity constant (K_i_^′^) of SY-1530 toward free BTK enzyme was comparable to the affinity constant (K_i_) of the BTK-ATP complex, thus indicating that SY-1530 and ATP bind BTK at different sites. The double reciprocal curve also showed that SY-1530 is a non-ATP competitive inhibitor (**Figure 1F**). Because the adverse effects of ibrutinib in clinical applications can be caused by off-target effects on other Tec family kinases and the epidermal growth factor receptor^34^, we performed HTRF assays to compare the effects of ibrutinib and SY-1530 on a series of target kinases. The IC_50_ values of SY-1530 on various targets were higher than those of ibrutinib (**Supplementary Table S1**). In addition, we tested the inhibitory potency of SY-1530 at a high concentration (1 µM) on 17 additional protein kinases. SY-1530 at the high concentration inhibited several kinases, including CSK, FGR, FYN, LCK, LYN A, and YES1 (**Supplementary Table S2**). These data demonstrate that SY-1530 is a highly selective BTK inhibitor that may be developed as a next-generation BTK inhibitor for further investigation.

### SY-1530 irreversibly binds BTK

**Figure 2:**
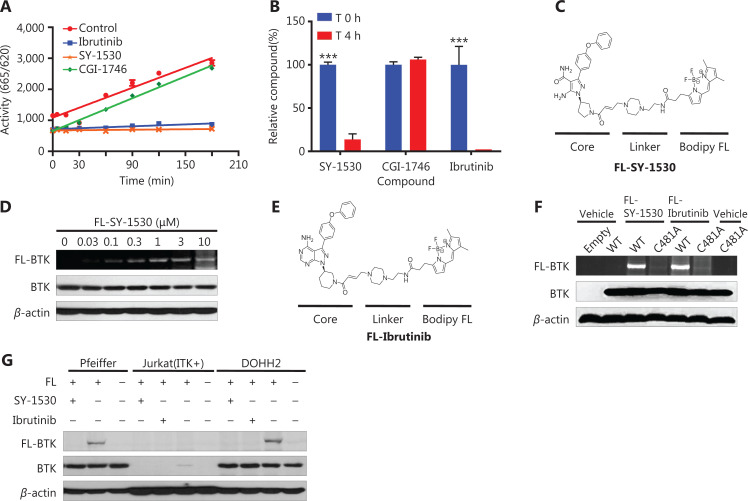
Irreversible targeting of BTK by SY-1530. (A) The compounds were incubated with BTK for 2 h, after which HTRF assays were performed with dilution of the mixture. The reactions were stopped at different time points. Data are shown as the mean ± SEM. (B) The content of the compounds was measured with LC-MS/MS after incubation with BTK for 4 h. Data are shown as the mean ± SEM (*n* = 3). Statistical analysis was performed with Student’s *t*-test (***P < 0.001). (C) Chemical structure of the Bodipy-FL fluorophore attached to the BTK inhibitor SY-1530 (FL-SY-1530). (D) DOHH2 cells were treated with various concentrations of FL-SY-1530 for 1 h, after which cells were lysed and analyzed by SDS-PAGE with the indicated antibodies and fluorescent gel scanning with a Typhoon imaging system. (E) Chemical structure of the labeled BTK inhibitor ibrutinib (FL-ibrutinib). (F) HEK293 cells expressing wild-type or C481A mutant BTK were treated with FL-SY-1530 or FL-ibrutinib for 1 h, and the cells were then lysed and analyzed. (G) Jurkat, Pfeiffer, and DOHH2 cells were treated with SY-1530 or ibrutinib for 1.5 h before incubation with FL-SY-1530 (FL). Cells were lysed and analyzed by Western blot and fluorescent gel scanning.

Next, we investigated the mechanism through which SY-1530 inhibits BTK. First, BTK was incubated with SY-1530 or other BTK inhibitors for 2 h, the reaction mixtures were diluted, and BTK activity was measured at different time points. The activity of BTK was restored by treatment with CGI-1746 (a reversible BTK inhibitor). In contrast, the activity of BTK did not recover in the SY-1530- or ibrutinib-treated groups, thus suggesting that SY-1530 might irreversibly bind BTK protein (**Figure 2A**). In addition, after incubation of BTK with the inhibitors for 4 h, we performed liquid chromatography-tandem mass spectrometry (LC-MS/MS) to detect the abundance of the inhibitors. Free SY-1530 or ibrutinib decreased by more than 80%, whereas CGI-1746 remained largely unchanged, thus supporting the covalent binding of SY-1530 to BTK (***P < 0.001) (**Figure 2B**). To further clarify the irreversible inhibition of SY-1530 on BTK *in vivo*, we synthesized fluorescently labeled SY-1530 (FL-SY-1530) for use in further studies (**Figure 2C**). We then treated DOHH2 cells, which endogenously express BTK, with various concentrations of FL-SY-1530 to examine whether FL-SY-1530 covalently bound BTK. An increased abundance of labeled BTK was observed with increasing FL-SY-1530 concentration (**Figure 2D**). Next, we sought to clarify whether SY-1530 covalently bound cysteine-481 of BTK. Another fluorescently labeled compound, the ibrutinib derivative FL-ibrutinib, was used as a positive control (**Figure 2E**). HEK293 cells expressing BTK wild-type or C481A mutant were treated with FL-SY-1530 or FL-ibrutinib. Both compounds bound the wild-type BTK but not the C481A mutant, thus indicating that BTK and SY-1530 formed a covalent complex (**Figure 2F**). In addition, to examine the selectivity of SY-1530 in cells, we used the Pfeiffer and DOHH2 B-cell lymphoma cell lines, which endogenously express BTK, and the Jurkat T-cell lymphoma cell line, which expresses ITK. After the cells were treated with FL-SY-1530, strongly labeled bands were observed for Pfeiffer and DOHH2 cells, but not Jurkat cells. Pretreatment with SY-1530 or ibrutinib significantly prevented the binding of labeled SY-1530 to BTK (**Figure 2G**). Together, these results suggested that SY-1530 selectively and irreversibly binds BTK.

### SY-1530 binds BTK in a dose-dependent and time-dependent manner

**Figure 3:**
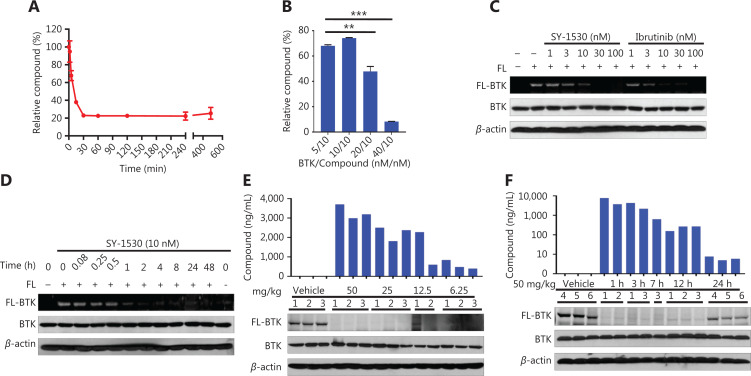
SY-1530 binds BTK in a dose-dependent and time-dependent manner. (A) The content of SY-1530 was measured with LC-MS/MS after incubation with BTK for the indicated time periods. Data are shown as the mean ± SEM. (B) SY-1530 was incubated with different concentrations of BTK, and LC-MS/MS was then performed to detect SY-1530 content. Data are shown as the mean ± SEM of 3 independent experiments. Statistical analysis was performed with Student’s *t*-test (**P < 0.01, ***P < 0.001). (C and D) DOHH2 cells were treated with different concentrations of SY-1530 or ibrutinib for 1 h (C) or 10 nM SY-1530 for the indicated time periods (D) and with 2.5 μM FL-SY-1530 for another hour. Cells were lysed and then analyzed by Western blot and fluorescent gel scanning. (E) Splenocyte extraction analysis was performed on normal BALB/c mice (6–8 weeks old) 3 h after oral administration of SY-1530 (50–6.25 mg/kg). The splenocytes were then incubated with labeled SY-1530 for 1 h, after which the total protein was extracted, and fluorescent gel scanning was performed with a Typhoon imaging system. The concentration of SY-1530 in the plasma was measured with LC-MS/MS. (F) Normal BALB/c mice (6–8 weeks old) were orally administered SY-1530 (50 mg/kg) and sacrificed at the indicated time points thereafter. Splenocyte extraction analysis was performed, and the splenocytes were incubated with labeled SY-1530 for 1 h. Cells were lysed and analyzed by Western blot and fluorescent gel scanning. The concentration of SY-1530 in the plasma was measured with LC-MS/MS.

Next, we performed LC-MS/MS assays to detect the content of SY-1530 after incubation with BTK for the indicated time periods. The covalent binding between SY-1530 and BTK kinase reached saturation after incubation for 30 min (**Figure 3A**). We also incubated SY-1530 with different concentrations of BTK and found that the percentage of free SY-1530 decreased as the concentration of BTK increased (**P < 0.01; ***P < 0.001) (**Figure 3B**). Furthermore, we treated DOHH2 cells with different concentrations of SY-1530 or ibrutinib for 1 h and with 2.5 µM FL-SY-1530 for another hour. FL-SY-1530 scarcely bound BTK when the concentration of SY-1530 or ibrutinib was 10 nM (**Figure 3C**). The binding of FL-SY-1530 to BTK decreased with increasing incubation time of BTK with SY-1530 (**Figure 3D**). To better understand the irreversible inhibition of SY-1530 under physiological conditions, we next extracted splenocytes from normal BALB/c mice after oral administration of SY-1530, incubated the cells with FL-SY-1530, and measured the concentration of SY-1530 in plasma. The fluorescently labeled BTK in mouse splenocytes significantly decreased with increasing SY-1530 dose, and the concentration of SY-1530 in the plasma was positively correlated with the oral dose (**Figure 3E**). Interestingly, after treatment with SY-1530 at 50 mg/kg, the exposure of SY-1530 in plasma gradually decreased with prolonged administration time. The BTK protein in splenocytes was still bound by SY-1530 at 12 h after administration. However, the exposure of SY-1530 in plasma decreased to a low level at 24 h after administration, and some BTK protein was released and bound the fluorescent label (**Figure 3F**). These results suggest that SY-1530 covalently binds BTK in a dose-dependent and time-dependent manner, and that the binding of SY-1530 to BTK *in vivo* has a direct positive correlation with its exposure in plasma.

### SY-1530 blocks B-cell receptor signaling and induces apoptosis

**Figure 4:**
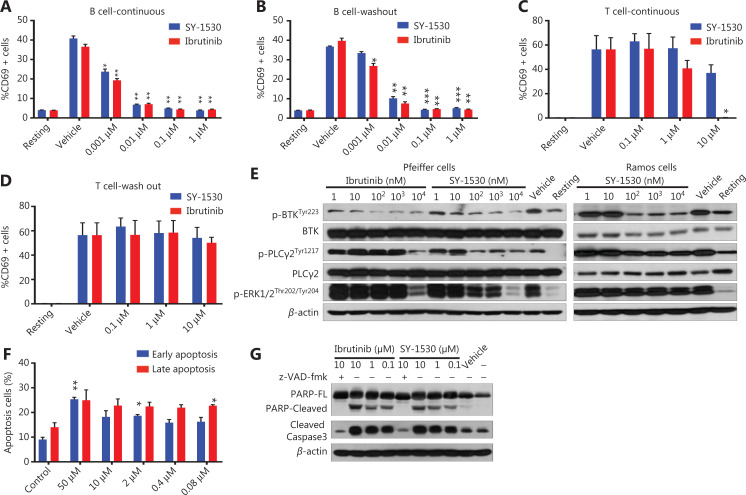
SY-1530 blocks B-cell receptor signaling and induces apoptosis. (A–D) Human B and T cells were purified and incubated with different concentrations of BTK inhibitors for 1 h. B cells were stimulated with 50 μg/mL anti-human IgM F(ab′)_2_, and T cells were stimulated with 50 μg/mL anti-CD3/CD28 beads overnight directly or after washout of BTK inhibitors before stimulation. The percentage of CD69 positive cells was analyzed by flow cytometry. Data are shown as the mean ± SEM (*n* = 3). Statistical analysis was performed with Student’s *t*-test (*P < 0.05, **P < 0.01, ***P < 0.001). (E) Pfeiffer cells and Romas cells were preincubated with different concentrations of compounds for 1 h, washed with PBS, and then stimulated with anti-IgM/anti-IgG (10 μg/mL) for 10 min. Cells were lysed and analyzed by Western blot with the indicated antibodies. (F) Pfeiffer cells were treated with various concentrations of SY-1530 for 24 h. The cells were stained with both PE-Annexin and 7-AAD and analyzed by flow cytometry. Data are shown as the mean ± SEM (*n* = 3). Statistical analysis was performed with Student’s *t*-test (*P < 0.05, **P < 0.01). (G) Pfeiffer cells were incubated with BTK inhibitors in the presence of z-VAD-fmk (caspase inhibitor) or DMSO for 24 h. Cells were lysed and analyzed with specific antibodies.

Previous studies have shown that BTK plays a crucial role in BCR signaling and is required for normal B-cell development, proliferation, and survival^9^. Because SY-1530 is an irreversible inhibitor of BTK that shows high selectivity for BTK *in vitro*, we hypothesized that SY-1530 might specifically inhibit B cell function. To examine this possibility, we first assessed the effect of SY-1530 on human peripheral B cells and T cells. Cells were treated with BTK inhibitors for 1 h and then stimulated as indicated. The expression of the lymphocyte activation marker CD69 was up-regulated after BCR stimulation by anti-IgM. However, this up-regulation of CD69 in B cells was completely abolished after continuous exposure to 10 nM SY-1530 or ibrutinib (*P < 0.05; **P < 0.01) (**Figure 4A**) and was also blocked even when the BTK inhibitors were washed out before BCR stimulation. Therefore, SY-1530 and ibrutinib irreversibly inhibit B cell activation (*P < 0.05; **P < 0.01; ***P < 0.001) (**Figure 4B**). In contrast, continuous exposure to 10 µM SY-1530 slightly inhibited, and that to ibrutinib significantly abrogated, CD69 up-regulation after T cell receptor stimulation (*P < 0.05) (**Figure 4C**), thus indicating that SY-1530 suppressed T cell stimulation much more weakly than ibrutinib. Moreover, after the washout of BTK inhibitors, the up-regulation of CD69 in T cells was not affected, thus suggesting that the inhibitory effects of SY-1530 and ibrutinib on T cell activation were reversible (**Figure 4D**). Therefore, in accordance with the selective inhibition of BTK, SY-1530 inhibited B cell activation rather than T cell activation. Next, to investigate whether SY-1530 blocked the BCR signaling pathway similarly to ibrutinib, we treated several B-cell lymphoma cell lines with a series of concentrations of inhibitors, washed them with PBS, and stimulated them with anti-IgM/anti-IgG. The BCR signaling pathway was activated after stimulation (**Figure 4E**, lane 11 and 12). As expected, the autophosphorylation of BTK, phosphorylation of PLCγ2, and phosphorylation of the downstream effectors ERK1/2 in Pfeiffer and Ramos cells were significantly inhibited by SY-1530 or ibrutinib (**Figure 4E**).

Abnormal activation of BCR signaling in B-cell malignancies leads to the resistance of tumor cells to apoptosis. To further explore the anti-tumor effect of SY-1530, we performed apoptosis assays by staining the cells with Annexin V and 7-AAD after treatment with SY-1530 for 24 h. SY-1530 induced early and late apoptosis in Pfeiffer cells (*P < 0.05; **P < 0.01) (**Figure 4F**). Cleavage of PARP, a DNA repair enzyme, is catalyzed by activated caspases during apoptosis^45^. To further confirm the effect of SY-1530 on apoptosis, we incubated Pfeiffer cells with the indicated inhibitors in the presence or absence of z-VAD-fmk (a pan-caspase inhibitor). Western blot analysis showed that cleaved PARP and caspase 3 increased in response to SY-1530 or ibrutinib treatment, and these effects were blocked by z-VAD-fmk addition (**Figure 4G**). Overall, our results suggested that SY-1530 blocks B-cell receptor signaling and induces caspase-dependent apoptosis.

### SY-1530 inhibits B-cell malignancies

**Figure 5:**
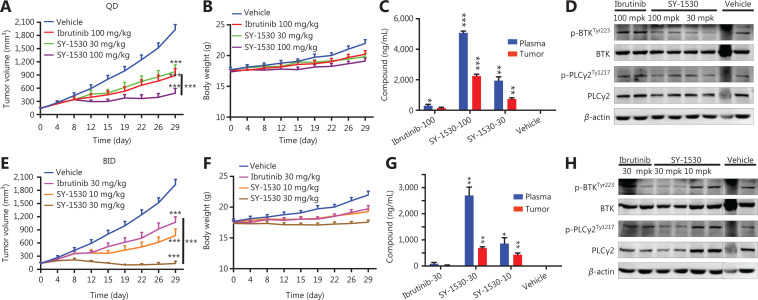
SY-1530 inhibits B-cell malignancy *in vivo*. (A–D) TMD8 cells were injected subcutaneously into SCID mice (6–8 weeks of age; 8 mice per group). SCID mice bearing TMD8 tumors were treated with SY-1530 (100 mg/kg or 30 mg/kg) or ibrutinib (100 mg/kg) once daily for 32 days. Tumor size (A) and body weight (B) were measured each week. On the last day, mice were sacrificed 2.5 h after administration. LC-MS/MS was performed to measure the concentration of SY-1530 in plasma (C), and tumor samples were collected and analyzed with Western blot (D). QD represents administration once daily. Data are shown as mean ± SEM. Statistical analysis was performed with two-way analysis of variance (*P < 0.05; **P < 0.01; ***P < 0.001). (E–H) SCID mice bearing TMD8 tumors were treated with SY-1530 (30 mg/kg or 10 mg/kg) or ibrutinib (30 mg/kg) twice daily for 32 days. Tumor size (E), body weight (F), SY-1530 concentration in the plasma (G), and protein expression in tumors (H) were measured. Data are shown as mean ± SEM. Statistical analysis was performed with two-way analysis of variance (*P < 0.05; **P < 0.01; ***P < 0.001).

To further determine whether SY-1530 inhibits B-cell malignancies, we first treated human B cells, T cells, and B-cell lymphoma cell lines with SY-1530 or ibrutinib *in vitro*. SY-1530 inhibited the proliferation of normal B cells and lymphoma cells, with IC_50_ values comparable to those of ibrutinib. In contrast, both SY-1530 and ibrutinib showed marginal inhibition of human T cell proliferation (**Supplementary Table S3**). Then, to explore the effects of SY-1530 on B-cell malignancies *in vivo*, we subcutaneously injected SCID mice with TMD8 cells and administered SY-1530 or ibrutinib once (QD) or twice (BID) per day for 29 days. In the QD groups, oral administration of SY-1530 resulted in a significant decrease in tumor volumes, and administration of SY-1530 at 30 mg/kg had anti-tumor activity comparable to that of ibrutinib at 100 mg/kg in TMD8 xenografts. Consequently, SY-1530 at 100 mg/kg showed better inhibitory potency on tumor growth than ibrutinib (***P < 0.001) (**Figure 5A**). Meanwhile, the bodyweights of mice were moderately changed, thus suggesting the toxicity of SY-1530 is limited (**Figure 5B**).

For pharmacokinetic/pharmacodynamic (PK/PD) studies, mice were sacrificed 2.5 h after administration on the last day. The concentrations of SY-1530 in the plasma were measured, and tumor samples were collected and subjected to Western blot analysis. The concentrations of SY-1530 in plasma were higher than those of ibrutinib and were positively correlated with the dose of SY-1530 and the anti-tumor activity (*P < 0.05; **P < 0.01; ***P < 0.001) (**Figure 5C**). Moreover, the autophosphorylation of BTK and the phosphorylation of PLCγ2 were impaired in the SY-1530 administration groups (**Figure 5D**).

To investigate whether BID administration was superior to QD administration, we subcutaneously injected SCID mice with TMD8 cells and treated them with SY-1530 (30 mg/kg or 10 mg/kg) or ibrutinib (30 mg/kg) BID. The tumor growth was dramatically lower in the groups treated with SY-1530 or ibrutinib (BID) than in the vehicle treated group (***P < 0.001). BID treatment with SY-1530 at 30 mg/kg showed better inhibitory potency on tumor growth than the same dose of ibrutinib (***P < 0.001) (**Figure 5E**). Moreover, the treatment with SY-1530 or ibrutinib (BID) induced a moderate decrease in mouse bodyweight (**Figure 5F**). The anti-tumor activity in the 30 mg/kg BID group was better than that in the 100 mg/kg QD group, and that in the 10 mg/kg BID group was better than that in the 30 mg/kg QD group (**Figure 5A and 5E**). In PK/PD studies, SY-1530 also suppressed the phosphorylation of BTK and PLCγ2 and showed better potency than ibrutinib in parallel experiments (*P < 0.05; **P < 0.01) (**Figure 5G and 5H**). More importantly, a 30 or 10 mg/kg BID dose of SY-1530 displayed a better effect on blocking BCR signaling pathway than a 100 or 30 mg/kg QD dose, in agreement with the *in vivo* anti-tumor activities. Thus, SY-1530 showed much better anti-tumor activity than ibrutinib. Moreover, BID administration performed better than QD administration in inhibiting BCR signaling and tumor growth *in vivo*. This information may be meaningful for clinical administration.

### SY-1530 inhibits canine B-cell lymphoma

**Figure 6:**
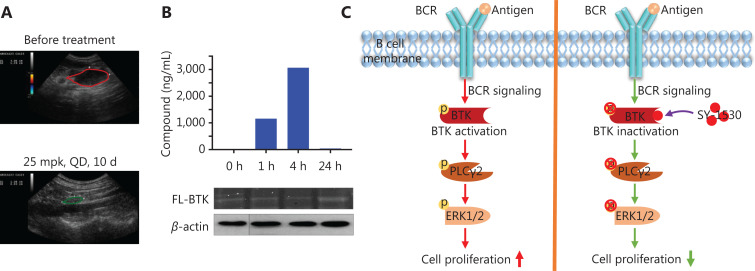
SY-1530 inhibits canine B-cell lymphoma. (A) Clinical response in spontaneous canine B-cell lymphoma was detected by abdominal ultrasonographic examination. (B) Targeting of BTK by SY-1530 in canine B-cell lymphoma was detected with Western blot and Typhoon fluorescence scanning. SY-1530 content was detected by LC-MS/MS analysis. (C) Schematic of how SY-1530 blocks the BCR signaling pathway. After antigen binding, BCR transduces activation signals into cells. BTK and its downstream kinases are then activated. SY-1530 abrogates the BCR signaling pathway through inhibiting BTK activity, thus inhibiting cell proliferation.

To further investigate the anti-lymphoma activity of SY-1530, we examined the effect of SY-1530 on naturally occurring B-cell lymphoma in canines, which shares common pathological features with B-cell lymphoma in humans. SY-1530 showed good anti-tumor effects in spontaneous canine B-cell lymphoma (**Table 1**). After treatment with SY-1530; 1 of 10 dogs showed a complete response, 3 showed a partial response, and 6 maintained stable disease. The subperitoneal lymph nodes completely disappeared in 1 dog after treatment with SY-1530 for 1 week (**Figure 6A**). In PK/PD studies, the irreversible binding of SY-1530 to BTK was detected in peripheral blood mononuclear cells, thus indicating that SY-1530 suppresses tumor growth through inhibiting BTK (**Figure 6B**).

**Table 1:** Summary of the effect of SY-1530 on canine B-cell lymphoma

Dog	Stage	Dose (mg/kg)	Outcome	Treatment interval (d)
01	IVb	20/35	SD/PR	21
02	IVa	20/30/40	CR	150
04	IVa	20/30/40	SD	21
06	IVa	20/30/40	SD	21
07	IVa	20/30	SD	14
08	IVa	35	SD	21
09	IVa	35	SD	21
10	IVa	35	PR	28
11	IIIa	35	PR	14
12	IVa	35	SD	21

CR, complete response; PR, partial response; PD, progressive disease; SD, stable disease.

## Discussion

In this study, our results revealed that SY-1530, a novel BTK inhibitor, has significant inhibitory activity in mouse xenograft models and spontaneous canine B-cell lymphoma. SY-1530 inhibits the phosphorylation of BTK and the activity of BTK downstream kinases such as PLCγ2 and ERK, thus blocking the BCR signaling pathway and suppressing tumor growth in B-cell malignancies (**Figure 6C**). SY-1530 also shows a higher selectivity and better anti-tumor effects than ibrutinib and therefore may be a promising drug for treating B-cell malignancies.

Ibrutinib is an orally active, first-in-class BTK inhibitor that acts through binding BTK. It selectively inhibits the autophosphorylation of BTK at tyrosine 223, with an IC_50_ of approximately 0.5 nM *in vitro*^25^. Despite being a promising therapy for B cell malignancies, ibrutinib still shows drug-dependent toxicity, which has been attributed to its off-target effects on other kinases^34,46–48^. Additionally, mutations in BTK can lead to the development of drug resistance in some patients. We found that SY-1530 had inhibitory effects on BTK activity comparable to those of ibrutinib but higher BTK specificity (**Figure 1A–1D**). SY-1530 inhibited BCR activation in B cells but did not affect T-cell receptor activation (**Figure 4A–4D**). Patients usually discontinue ibrutinib treatment because of adverse effects. However, SY-1530 alleviates these adverse effects, including bleeding and interstitial lung disease, possibly because of its high BTK specificity. However, we cannot exclude the possibility of inhibitory effects of SY-1530 on some Src family kinases.

SY-1530 showed better anti-tumor activity than ibrutinib by blocking the activation of the BCR signaling pathway in TMD8 tumor xenograft models. It also had higher drug concentration in plasma and tumor than ibrutinib (**Figure 5**). The high drug level of SY-1530 might induce a better clinical response in patients. The BCR signaling pathway has been demonstrated to play important roles in autoimmune disorders and some B-cell lymphomas^20,49,50^. However, the results in tumor xenograft studies have shown limited correlation to B-cell lymphoma growth *in vivo*. Therefore, we used a model of canine B-cell lymphoma to investigate SY-1530 in animals and found that SY-1530 was efficacious in treating spontaneous canine B-cell lymphoma (**Figure 6, Table 1**). These results indicated that SY-1530 is a potent drug for the treatment of B-cell malignancies. In addition, it has a unique chemical structure, and showed an excellent absorption, distribution, metabolism, excretion, and toxicity (ADMET) profile in preclinical studies (**Supplementary Figures S1–S3 and Supplementary Tables S4–S9**). Further clinical trials should be performed to evaluate the efficacy, safety, pharmacokinetics, and pharmacodynamics of SY-1530 in patients with B-cell lymphomas. Furthermore, other BTK inhibitors are under evaluation for the treatment of autoimmune diseases in which BTK has known functions. Therefore, investigations of SY-1530 could also be expanded to include autoimmune diseases in the future.

The applications of ibrutinib in clinical settings have been restricted because of the unfavorable effects and drug resistance. Second-generation BTK inhibitors, including acalabrutinib, zanubrutinib, and tirabrutinib, are more potent and selective than ibrutinib^34,40,51,52^. Although these drugs have high clinical and preclinical efficacy, they exhibit similar toxic effects to those of ibrutinib. The third-generation BTK inhibitors that effectively target wild-type and Cys481 mutant forms of BTK are in early clinical trials. ARQ-531 is a reversible and non-covalent BTK inhibitor in phase I clinical development for the treatment of patients with relapsed or refractory hematologic malignancies^43,53^. Clinical trials of LOXO-305 and SNS-062, which are also inhibitors of BTK and the BTK^C481S^ mutant, are currently underway^43,54–56^. SY-1530 is in phase I clinical trials for multiple B-cell malignancies, including CLL, MCL, diffuse large B-cell lymphoma, and WM. To bring more clinical benefits to patients, ascertaining the potential advantages and disadvantages of SY-1530 and the next-generation BTK inhibitors will be important in future clinical investigations.

## Conclusions

Here, we report that SY-1530, a novel BTK inhibitor, blocks the BCR signaling pathway through the down-regulation of BTK activity. SY-1530 specifically inhibits B-cell activation rather than T-cell activation, and thus decreases the off-target effects and related toxicity. Trials of SY-1530 are currently underway in patients with multiple B-cell malignancies to evaluate its safety and clinical activity. In conclusion, SY-1530 is a promising therapeutic agent for the treatment of B-cell malignancies.
