# Supplementary material for: SY-1530, a highly selective BTK inhibitor, effectively treats B-cell malignancies by blocking B-cell activation
Source: Cancer Biol Med. 2021 Jul 15;19(7):995–1007. doi: 10.20892/j.issn.2095-3941.2020.0291 (PMC9334755; doi:10.20892/j.issn.2095-3941.2020.0291)
Supplement: Supplementary file 1 [file cbm-19-995-s001.pdf]

Supplementary materials

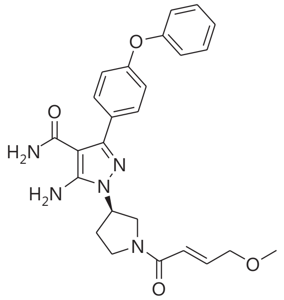

Figure S1 The chemical structure of SY-1530.

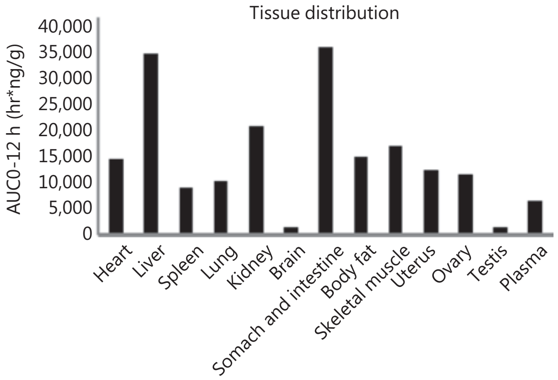

Figure S2 The distribution profile of SY-1530. Tissue distribution of SY-1530 in rats was conducted with ^{14}C-SY1530 after oral administration.

Table S1 The effect of SY-1530 or ibrutinib on the activity of various kinases

Targets	IC_{50} (nM)		Ratio
	Ibrutinib	SY-1530	
ITK	75.3	>1000	>13
TEC	7.89	10.4	1.3
EGFR	1.6	360	225
JAK3	368	>10000	>27
HER2	3.9	>10000	>2500
BMX	0.8*	7.51	9.4

*From the literature.

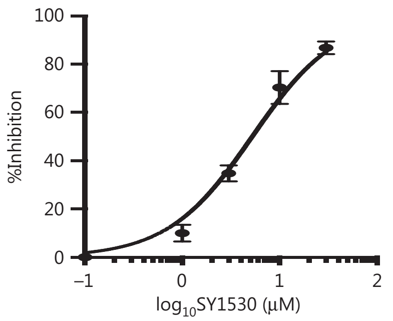

hERG inhibition

Compound	Maximum concentration (μM)	Inhibition rate of maximum concentration	IC_{50} (μM)
SY-1530	30	86.75 ± 2.61%	5.41

Figure S3 The effect of SY-1530 on the hERG. The IC_{50} of SY-1530 on the hERG was measured with HTRF assays.

Table S2 The inhibitory effect of SY-1530 on the activity of 17 protein kinases

Kinase	Inhibition (%)	Kinase	Inhibition (%)
BLK	53	HCK	67
CSK	84	LCK	94
FGFR3	25	LYN A	87
FGR	96	PTK6	92
FLT1	33	RET	57
FLT3	68	TXK	73
FRK	72	YES1	94
FYN	87	EPHA3	−2
ABL1	8		

Red color, ≥ 80% inhibition; blue color, < 40% inhibition; white color, 40%–80% inhibition.

Table S3 The effect of SY1530 on the growth of human B cells, T cells, and B-cell lymphoma

Cell line	IC_{50} (nM)	
	SY-1530	Ibrutinib
TMD8	15.6	17.3
Pfeiffer	71.4	45.9
B cell	1.05	0.85
T cell	>10000	1000–10000

Table S4 The metabolism of SY-1530 in rats

	Rat PK			
	IV	Oral		
Dose (mg/kg)	5	30	100	300
t½ (h)	3.08	4.79	3.08	3.9
Tmax (h)		3.33	1.75	5.25
Cmax (ng/mL)		658	1085	1129
AUC0–∞ (ng•h/mL)	4112	4460	6842	9257
CL (mL/h/kg)	1374			
Vss (mL/kg)	1835			
F (%)		18.1	8.32	3.75

Table S5 The metabolism of SY-1530 in beagle dogs

	Beagle dog PK			
	IV	Oral		
Dose (mg/kg)	3	10	30	100
t½ (h)	1.44	3.85	5.33	5.03
Tmax (h)		1.08	0.917	1.25
Cmax (ng/mL)		1039	1572	2127
AUC0–∞ (ng•h/mL)	5458	4411	6365	9095
CL (mL/h/kg)	559			
Vss (mL/kg)	857			
F (%)		24.2	11.7	5.00

Table S6 The extent of SY-1530 binding to plasma protein

Species	Plasma protein binding (%)
Rat	95.0
Mouse	94.8
Human	95.6
Dog	95.5
Monkey	95.3

Table S7 Absorption of orally administered SY-1530

Compound	Caco-2 permeability assay			
	Papp ($\times 10^{-6}$ cm/s)			
	Concentration (μM)	AP→BL	BL→AP	Ratio*
CT-1530	0.1	7.29	18.0	2.46
	1	6.62	17.1	2.58
	10	6.09	17.8	2.92

*Ratio = Papp BL→AP/Papp AP→BL, propranolol reference value: 28.6×10^{-6} cm/s, atenolol reference value: 0.455×10^{-6} cm/s

Table S8 Inhibition of CYP isozymes by SY-1530

	CYP1A2	CYP2B6	CYP2C8	CYP2C9	CYP2C19	CYP2D6	CYP3A4 (Mid)	CYP3A4 (Tes)
IC_{50} (μM)	>25	>25	>25	>25	23.8	>25	>25	13.7

Table S9 Preclinical safety of SY-1530

General toxicity				
Group	Single-dose toxicity		Multiple-dose toxicity	
Animal	Rat	Beagle dog	Rat	Beagle dog
MTD (mg/kg)	2000	1000	300 (male), 175 (female)	160
